# Long-Term Survival After Complications Following Major Abdominal Surgery

**DOI:** 10.1007/s11605-016-3084-4

**Published:** 2016-02-08

**Authors:** Jennifer Straatman, Miguel A. Cuesta, Elly S. M. de Lange – de Klerk, Donald L. van der Peet

**Affiliations:** Department of Surgery, VU University Medical Center, De Boelelaan 1117, ZH 7F020, 1081 HV Amsterdam, The Netherlands; Department of Epidemiology and Biostatistics, VU University Medical Center, van der Boechhorststraat 7, 1081 BT Amsterdam, The Netherlands

**Keywords:** Postoperative complications, Survival, Major abdominal surgery

## Abstract

**Introduction:**

Postoperative complications have been associated with decreased long-term survival in cardiac, orthopedic, and vascular surgery. For major abdominal surgery research, conflicting evidence is reported in smaller studies. The aim of this study was to assess the effect of complications on long-term survival in major abdominal surgery.

**Material and Methods:**

An observational cohort study was conducted of 861 consecutive patients that underwent major abdominal surgery between January 2009 and March 2014, with prospective assessment of the survival status. The effect of postoperative complications on survival was assessed.

**Results:**

Postoperative complications were associated with decreased survival, even after applying correction for 30-day mortality (*p* < 0.001). Stratified Cox regression analysis depicted postoperative complications to be an important predictor for survival in upper gastrointestinal and female hepatopancreaticobiliary patients. Correction was applied for age, gender, BMI, ASA classification, radicality, and positive lymph node status.

**Conclusion:**

These results further indicate the importance of prevention and early diagnosis and treatment of complications. Etiological factors are believed to be both sustained levels of inflammatory markers, as well as attenuated immune response in malignancy with subsequent cancer cell seeding. Future research should aim to prevent and early diagnose postoperative complications to prevent morbidity and mortality not only in the early postoperative phase, but also in the long term.

## Introduction

Over 20,000 major abdominal surgical procedures, consisting of digestive resections with reconstruction via anastomosis and/or stoma are performed each year in the Netherlands.^1^ Twenty percent of these patients suffer a major complication in their postoperative course, necessitating invasive treatment such as percutaneous drainage, reoperation, and admission to the intensive care unit (ICU).^2^

Literature has shown postoperative complications to affect long-term survival in cardiac surgery, orthopedic surgery, and vascular surgery, regardless of preoperative risk assessment.^3^ An important finding, which has further supported improvement in postoperative processes and quality control.^4^

The effect of postoperative complications on long-term survival in patients that undergo major abdominal surgery has not yet been fully described. One study found increased mortality rates in patients who suffered complications after colectomy, but the follow-up period was only 60 days.^5^ Conflicting results from smaller studies are reported following esophageal resection^6^,^7^ and hepatopancreaticobiliary resections.^8^,^9^ Results from these studies cannot be pooled since different patient groups and different definitions for postoperative complications are used, and no correction was applied for primary deaths caused by complications.

In order to necessitate and initiate postoperative quality control algorithms in major abdominal surgery, it is imperative that the current effect of postoperative complications on long-term survival is depicted. This analysis should be conducted with regard to the patient characteristics and take into account possible bias. The use of a more reliable and reproducible grading of complications is proposed, the classification system of Clavien-Dindo, which grades complications according to the necessitated treatment.^10^,^11^

The aim of this study is to assess the effect of postoperative complications on long-term survival in patients that underwent major abdominal surgery. In major abdominal surgery, do major postoperative complications affect long-term survival in comparison to patients with an uncomplicated postoperative course after 1-year follow-up?

## Methods

An observational cohort study was conducted with prospective assessment of survival. Including all consecutive patients who underwent major abdominal surgery, between January 2009 and May 2014, in the VU University Medical Center, Amsterdam, the Netherlands.

### Definitions and Parameters

Major abdominal surgery was defined as all digestive resections with reconstruction via anastomosis and/or stoma. For instance, cholecystectomy was not considered major abdominal surgery as no anastomosis or stoma is performed.

Both benign and malignant indications for surgery are included and both conventional open and minimally invasive techniques were included.

Postoperative complications were graded according to the Clavien-Dindo classification, which grades complications according to the necessitated treatment.^10^,^11^ Since it is particularly important to distinguish complications that require surgical, endoscopic, or radiological interventions, Clavien-Dindo classification was modified to two groups. Group I consisted of grade I and grade II complications and are classified as “minor complications,” while group II consisted of grade III, IV, and V complications and are classified as “major complications.”

Recorded clinical parameters included baseline characteristics, indication for surgery, operative procedures, postoperative clinical parameters and recovery, and postoperative complications. Baseline characteristics such as height, weight, comorbid diseases and medication are routinely assessed at preoperative workup. Smoking history was determined as smoking in the last year, defined as a person who smokes on a daily basis.

All patients received perioperative prophylactic intravenous antibiotics, usually consisting of one perioperative gift of antibiotics, and thromboembolic prophylaxis according to local protocol, consisting of low-molecular-weight heparin during the entire duration of admission. Treatments of major complications were classified as reoperations, radiological interventions such as percutaneous drainage and intensive care admission. All patients had routine follow-up at the outpatient clinic at least once at 1–3 months after admission to assess patient status and in order to monitor for complications, which are registered in the hospital’s complication database.

Follow-up data concerning the survival status of patients could be obtained in a prospective manner by the outpatient policlinic, and if necessary using the Municipal Personal Records Database. In the case of no proper documentation on follow-up, the general practitioner (GP) was directly contacted about the survival status of a determined patient.

### Statistics

Statistical analysis was conducted in SPSS version 21.0 (SPSS Inc., Chicago, IL, USA). Continuous variables with normal distributions were presented as means and standard deviations. Medians and interquartile ranges were used as central tendency for continuous variables with nonnormal distributions. Categorical data were expressed with percentage frequencies and compared using the chi-squared test. A two-sided *p* value of <0.05 was considered statistically significant.

Comparison of survival between patients with major, minor, or no complications was conducted using Kaplan-Meier curves with a log-rank test. Confounding and effect modification were assessed using the Cox regression technique. Correction was applied for comorbidities, malignancy, and type of operation (upper gastrointestinal, hepatopancreaticobilliary or colorectal).

This study was assessed and approved by the VU University Medical Center, Medical Ethics Committee. Informed consent was waived due to the observational nature of the study.

## Results

Eight hundred sixty-one patients underwent elective major abdominal surgery in the VU University Medical Center, Amsterdam, the Netherlands, between January 2009 and March 2014. Two hundred patients (23.2 %) underwent upper gastrointestinal surgery, consisting of 145 patients undergoing esophagectomy and 55 patients who underwent gastrectomy for cancer. One hundred three patients (12 %) underwent hepatopancreaticobiliary surgery, which included pancreaticoduodenectomy (*n* = 83) and partial liver resections (*n* = 16), or reconstruction of the biliary tree (*n* = 4). Five hundred fifty-eight patients (64.8 %) underwent colorectal surgery, including (partial) colectomies (*n* = 401) and rectal resections (*n* = 157) mostly for cancer.

### Postoperative Complications

Postoperative complications were recorded in 365 patients (42.4 %). A major complication was recorded in 211 patients (24.5 %), as graded according to the Clavien-Dindo classification grades III and up. These complications required invasive treatment, such as percutaneous drainage, reoperation, or intensive care management. An overview of baseline characteristics for the different classes of complications is depicted in Table 1. Patients with minor complications were significantly older compared to patients without complications or major complications (*p* < 0.001). Patients who underwent upper gastrointestinal (upper GI) surgery had an uncomplicated postoperative course less often compared to hepatopancreaticobilliary (HPB) and colorectal surgery (*p* < 0.01). An overview of postoperative complications is depicted in Table 2.

**Table 1:** Baseline characteristics

Parameter	Uncomplicated	Minor complication	Major complication	*p* value
Patients (*n*)	496 (57.6 %)	154 (17.9 %)	211 (24.5 %)	
Gender, male (%)	264 (53.2 %)	100 (64.9 %)	123 (58.3 %)	0.032
Age (years) (mean ± SD)^a^	61.2 ± 15	66.9 ± 13.5	61.8 ± 14.2	<0.001
Body mass index (BMI), mean ± SD	25.2 ± 4.6	25.3 ± 4.8	25 ± 4.3	0.602
ASA classification^b^				
I	77 (15.7 %)	12 (7.8 %)	20 (9.7 %)	0.024
II	313 (63.56 %)	100 (65.4 %)	125 (60.4 %)	
III	91 (18.5 %)	37 (24.2 %)	53 (25.6 %)	
IV	11 (2.2 %)	4 (2.6 %)	9 (4.3 %)	
Comorbid disorders	228 (46 %)	88 (57 %)	102 (48.3 %)	0.053
Smoking^b^	103 (20.9 %)	37 (24.3 %)	65 (31.2 %)	0.014
Operative details				
Indication				
Malignant	361 (72.8 %)	122 (79.2 %)	156 (73.6 %)	0.278
Benign	135 (27.2 %)	32 (20.8 %)	55 (26.1 %)	
Operation type^c^				
Upper GI	76 (15.3 %)	51 (33.1 %)	73 (34.6 %)	0.001
HPB	52 (10.5 %)	30 (19.5 %)	21 (10 %)	
Colorectal	368 (74.2 %)	73 (47.4 %)	117 (55.5 %)	
Minimally invasive	287 (56 %)	77 (50 %)	119 (56.4 %)	0.379
Conversion	27 (9.7 %)	13 (16.9 %)	18 (15.1 %)	0.127
Anastomosis	410 (82.7 %)	129 (83.8 %)	180 (85.3 %)	0.683
Stoma	113 (22.8 %)	36 (23.4 %)	43 (20.4 %)	0.734
Protective stoma	38 (9.3 %)	12 (9.3 %)	13 (7.2 %)	0.7
Duration of surgery (min ± SD)^d^	201 ± 91	238 ± 88	239 ± 101	0.001
Hospital stay (days (IQR))	7 (5–10)	13 (9–16)	21 (14–41)	0.001
ICU management (days (IQR))	1 (1–2)	1 (1–3)	6 (2–23)	0.001
Thirty-day mortality			11	

^a^Post hoc Bonferoni analysis revealed that age was significantly higher in patients with minor complications

^b^Post hoc Bonferoni analysis revealed no statistically significant differences between the three groups

^c^Post hoc Bonferoni analysis depicted less uncomplicated cases in upper GI

^d^Post hoc Bonferoni analysis showed duration of surgery to be shorter in uncomplicated cases

**Table 2:** Overview of postoperative complications after major abdominal surgery, stratified for type of surgery

Complication	Upper GI	Percent	HPB	Percent	Colorectal	Percent	*p* value
Patients (*n*)	200		103		558		0.001
Minor complication	51	25.5 %	30	29.1 %	73	13.1 %	
Major complication	73	36.5 %	21	20.4 %	117	21.0 %	
Postoperative bleeding	4	2.0 %	6	5.8 %	10	1.8 %	0.042
Surgery	4		4		6		
Transfusion	–		–		2		
Anastomotic leak	24	12.0 %	10	9.7 %	41	7.3 %	0.125
Surgery	11		7		39		
Percutaneous drainage	5		3		2		
Stent	8		–		–		
Abscess	19	9.5 %	9	8.7 %	49	8.8 %	0.808
Surgery	7		2		26		
Percutaneous drainage	11		7		23		
Perforate	6		1		12		0.517
Surgery	5		1		11		
Percutaneous drainage	–		–		1		
Stent	1		–		–		
Wound/stoma problem	10	5.0 %	11	10.7 %	41	7.3 %	0.184
Infection requiring surgery	1		1				
Fascial dehiscence	–		–		3		
Nonabdominal							
Pneumonia	56	28.0 %	9	8.7 %	33	5.9 %	0.001
Cardiac complications	8	3.0 %	3	2.9 %	17	3.0 %	0.001

### Thirty-Day Follow-Up

Eleven patients died within 30-day follow-up, being five patients after upper GI surgery (2.5 %), one patient after HPB surgery (1 %) and five patients after colorectal surgery (1 %) (*p* = 0.087). After upper GI surgery, four patients died following severe sepsis of which three patients had an anastomotic leak without clinical improvement after (re)intervention and one patient developed tracheal-esophageal fistula with severe mediastinitis. Finally, one patient had a leakage of the feeding naso-jejunal tube with subsequent mediastinitis and respiratory insufficiency.

One patient died following extensive HPB surgery, following massive postoperative bleeding from the gastroduodenal and superior mesenteric artery, which were dissected during the primary operation in order to obtain radicality.

Five patients died following colorectal surgery. Two patients developed severe sepsis with multi organ failure. Of these patients one patient died following anastomotic leak and another one following multiple perforations, which existed after extensive adhesiolysis. One patient died following severe myocardial infarction. Finally, one patient suffered from cerebrovascular ischemia with severe paresis, along with respiratory insufficiency following pneumonia.

Four patients of the cohort were lost to follow-up after their admission, being patients who lived in the Netherlands temporarily and continued treatment in their country of origin.

### Long-Term Survival

**Fig. 1:**
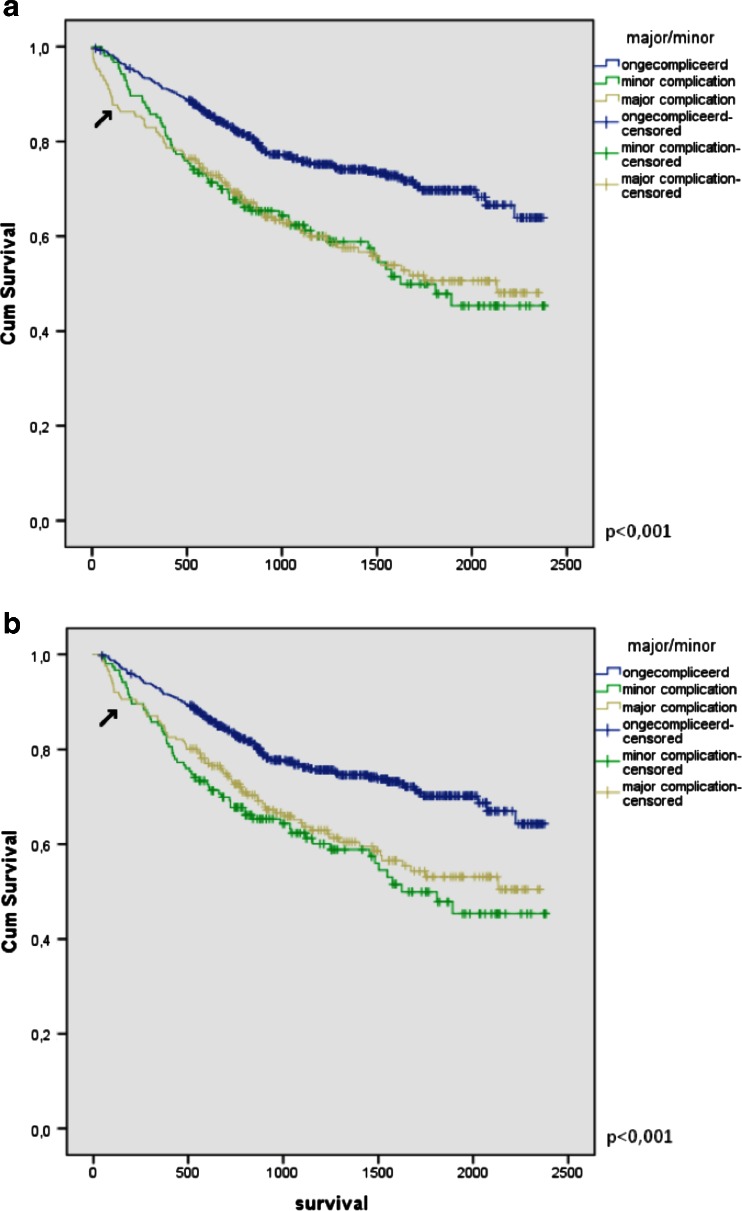
**a** Kaplan-Meier curve stratified for patients with no, minor or major complications. **b** Kaplan-Meier curve excluding patients with less than 30-day follow-up. *Arrow* indicates differences in curve for major complications

Average follow-up time was 1126 days (range 36–2381). Differences in survival rates were assessed using the Kaplan-Meier curve and log-rank test. With a mean survival of 1861 days (95 % CI 1784–1938) in uncomplicated patients, versus 1508 days (95 % CI 1353–1664) in patients with minor complications and 1500 days (95 % CI 1368–1633) in patients with major complications, patients with postoperative complications have worse survival (*p* < 0.001). Since grade V of the Clavien-Dindo classification entails death of the patient all patients with less than 30-day follow-up or death, as described above, were removed in the second analysis, results remained similar. Both graphs are depicted in Fig. 1.

**Fig. 2:**
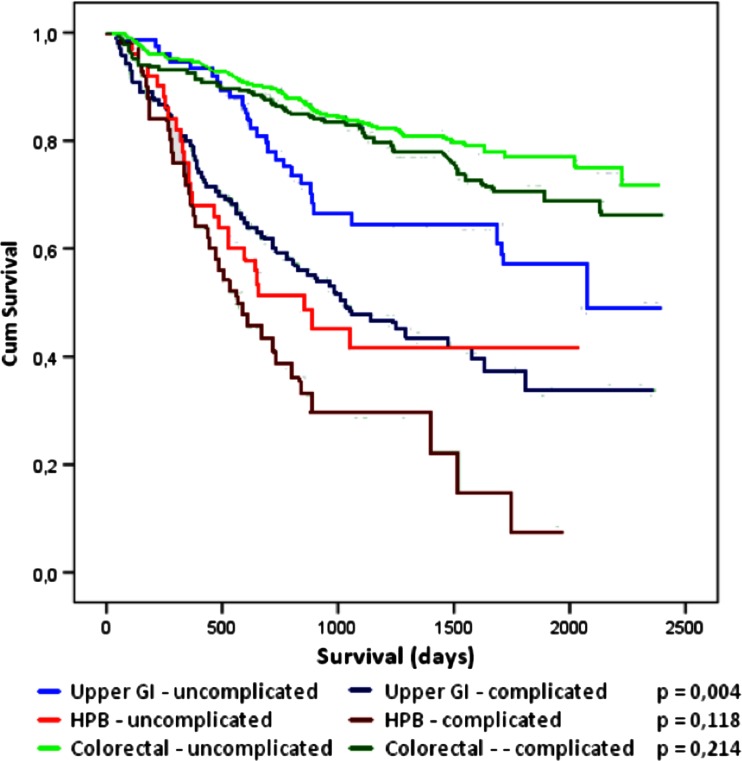
Stratified analysis of survival in uncomplicated and complicated cases in upper GI, HPB, and colorectal surgery. *p* Values depicted for log-rank test in each group

With different median survival rates reported for the different organ groups, stratified analysis was conducted for type of surgery, being upper GI, HPB, or colorectal. Postoperative complications only decreased long-term survival in upper GI surgery (*p* = 0.004). Survival for the three surgical groups is depicted in Fig. 2.

### Cox Regression

Cox regression analysis was performed stratified for the three organ groups. In each group, the following parameters were assessed: postoperative complications, age, gender, ASA classification, comorbidity, BMI, preoperative weight loss, minimally invasive surgery, radicality of the procedure, and positive lymph nodes. Each parameter was assessed separately for confounding and effect modification. In HPB, gender was found to be an effect modifier; hence, analysis was stratified for gender. Next, a backward stepwise logistic model was run in each surgical group to identify parameters that influence long-term survival.

In upper GI patients, postoperative complications (hazard ratio (HR) 2.2 (95 % CI 1.3–3.7)), preoperative weight loss (HR 1.9 (95 % CI 1.2–2.9)) and positive lymph nodes in pathology (HR 2.7 (95 % CI 2.7–4.3) were found to be predictors for worse survival outcomes. Minimally invasive surgery had a protective effect (HR 0.6 (95 % CI 0.4–0.9)), with the overall model *p* < 0.001.

In HPB, surgery analysis was stratified for gender. In male HPB patients, the final model included increasing age (HR 1.04 (95 % CI 0.99–1.088)), preoperative weight loss (HR 3.4 (95 % CI 1.3–9.0) and positive lymph nodes (HR 3.8 (95 % CI 1.2–12.2) were predictive of decreased survival rates. In female HPB patients, preoperative weight loss (HR 3.0 (95 % CI 1.2–7.7) and postoperative complications (HR 3.4 (95 % CI 1.4–7.9) were found to be significant predictors. The overall model had a significance level of *p* < 0.001.

In colorectal surgery, increasing age (HR 1.02 (95 % CI 1.0–1.04)), ASA classification (*p* = 0.016), preoperative weight loss (HR 1.7 (95 % CI 1.1–2.8)), and positive lymph nodes (HR 1.8 (95 % CI 1.2–2.6)) were predictive of decreased survival. Minimally invasive surgery had a protective effect (HR 0.5 (95 % CI 0.3–0.8), with overall model *p* < 0.001.

## Discussion

The article describes long-term follow-up after major abdominal surgery, with a follow-up of up to 5 years postoperatively. Long-term follow-up was affected by postoperative complications in the overall analysis. When stratified for different types of surgery, survival is mainly affected by postoperative complications in upper GI and female HPB patients. These results hold even when correction is applied for patients who survived less than 30 days postoperatively.

Previous studies have described similar results in other surgical patient groups. Khuri et al. using the National Surgical Quality Improvement Program (NSQIP) database, determined postoperative complications to be a predictor for worse survival in major surgery patients, including vascular, abdominal, pulmonary, and orthopedic procedures.^3^ All complications were grouped in this study and exact definitions omitted. A hazard ratio of 1.4 (95 % CI 1.2–1.6) was described for postoperative complications occurring within 30 days following pulmonary resections for cancer.^12^ In cardiac surgery, the length of intensive care stay was linked to worse survival rates.^13^ Previous studies only included colectomies and cholecystectomies.^3^,^14^ The here presented study depicts survival rates in all types of major abdominal surgery, including upper gastrointestinal, hepatopancreaticobiliary, and colorectal surgery and including only major procedures with resection and reconstruction with anastomosis and/or stoma.

Long-term survival is mainly affected by postoperative complications in the group of patients that underwent upper gastrointestinal surgery. A possible explanation may be sought in surgical site complications leading up to mediastinitis in this patient group. Mediastinits has previously been associated with decreased long-term survival in cardiac surgery patients.^15^ Additionally, anastomotic leakage following esophageal resection is an independent predictor for benign anastomotic strictures, which may lead to poor nutritional status and thereby affect long term survival.^16^

In hepatopancreaticobiliary surgery, complications affected long-term survival mainly in women. It should be noted that the HPB group was the smallest group in this cohort and effect modification caused by gender further led to assessment of even smaller groups. Although differences in survival for gender have been reported previously and may be related to differences in incidence,^17^ the results of the Cox regression should be interpreted with care.

Several pathways are described which may describe why postoperative complications affect long-term survival. In patients with postoperative morbidity, including both infective and noninfective complications, sustained levels of inflammatory markers are measured, such as C-reactive protein and procalcitonin.^2^,^18^ Prolonged inflammation is associated with the development of chronic cardiovascular and neurological disorders.^19^,^20^ In a later phase, these disorders may account for differences in mortality rates as seen in our analysis.

Other research focused on long-term outcomes in cancer patients. Surgical stress activates neuroendocrine and immunological pathways that suppress the immune response, which is associated with growth of residual malignant cells in cancer patients.^21^ Postoperative complications further amplify the stress response, along with critical illness, sepsis, and blood transfusions, further increasing cancer cell seeding in these patients.^22^,^23^ Subsequently, leading up to decreased rates of survival and disease-free survival. The Municipal Personal Records Database does not hold information on cause of death or malignancy status as it is a nonmedical registry. Hence, disease-free survival and progression-free survival could not be assessed in this cohort.

The study is limited by its observational nature. Especially with minimally invasive surgery, which was found to be a protective factor in upper GI and colorectal surgery, selection bias might affect results, with primary open resections being performed in patients with severe comorbid conditions or requiring extensive resections for larger masses. Although extensive testing was conducted in order to identify confounders and effect modifiers, it should be noted that variables which were not recorded in this dataset may also confound results.^24^

Correction was applied for patients who died within 30 days following surgery. Toner et al. proposed that bias might still occur with patients that died just after the 30-day follow-up. Analysis with patients in our sample corrected for 90-day follow-up depicted a similar Kaplan-Meier curve with log-rank test *p* < 0.001.

The here presented study further assessed the effect of postoperative complications on long-term survival in major abdominal surgery and found survival to be affected in patients that undergo upper GI surgery and female patients that undergo HPB surgery.

Survival may further be affected since postoperative adjuvant therapy may be delayed or not even initiated at all.^25^

Future research should aim to assess quality control algorithms in order to predict, in an early phase, postoperative complications. Early diagnosis and treatment have been shown to improve short-term outcomes regarding morbidity and mortality and may further improve long-term survival.^26^,^27^ A prospective clinical trial is currently underway from our department, assessing early diagnosis and treatment with standardized C-reactive protein measurements with additional imaging if CRP exceeds the predefined cutoff of 140 mg/L from postoperative day 3.^28^

In conclusion, postoperative complications affect long-term survival in major abdominal surgery, especially in upper GI and female HPB patients, further indicating the importance of prevention and early diagnosis and treatment of complications. Etiology is contributed to both sustained levels of inflammatory markers, as well as an attenuated immune response with subsequent cancer cell seeding. Future research should aim to prevent and early diagnose postoperative complications to prevent morbidity and mortality not only in the early postoperative phase but also in the long run.
